# Understanding stress from a cultural perspective: the role of self-construal

**DOI:** 10.3389/fpsyg.2026.1826049

**Published:** 2026-09-22

**Authors:** Xiang Hu, Yang He, Jiahao Luo, Zhenni Wei, Zhunzhun Liu, Juan Yang

**Affiliations:** 1 The Key Research Institute of Chongqing for Curriculum & Instruction, School of Education, Chongqing Normal University, Chongqing, China; 2 Chongqing Key Laboratory of Philosophy and Social Sciences for Intelligent Rehabilitation and Education of Children with Special Needs, Chongqing Normal University, Chongqing, China; 3 Student Affairs Office, Shangluo University, Shangluo, Shanxi, China; 4 Faculty of Psychology, Southwest University, Chongqing, China; Key Laboratory of Cognition and Personality, Ministry of Education, Southwest University, Chongqing, China; 5 Nursing School, Wuxi Taihu University, Wuxi, Jiangsu, China

**Keywords:** acute stress, chronic stress, culture, post-traumatic stress disorder, self-construal, stress

## Abstract

Stress is an adaptive psychophysiological process that plays an important role in human survival and adjustment. Increasing evidence suggests that stress responses are shaped not only by environmental demands but also by cultural and self-related psychological processes. Self-construal, which refers to how individuals define themselves in relation to others, has been widely regarded as an important mechanism linking culture to cognition, emotion, motivation, and behavior. However, its role in stress-related processes has not been systematically synthesized. The current narrative review integrates evidence from cultural psychology, stress research, neuroscience and PTSD literature to assess the impact of self-construal on stress perception, emotional regulation, coping, stress recovery and vulnerability to stress-related disorders. Existing evidence suggests that interdependent self-construal (InterSC) is associated with increased sensitivity to social-evaluative settings, more acute stress reactions, and higher chronic stress levels. Concurrently, InterSC may also assist a faster recovery from stress and better sensitivity to social support in specific cultural circumstances. Evidence on independent self-construal (IndeSC) is relatively scarce, but available findings suggest that autonomy, personal objectives, and self-consistency may serve as relevant stress-related themes among individuals with high IndeSC. Thus, self-construal provides a valuable framework for understanding the ways that culturally molded self-processes influence stress responses, adaptability, and mental health outcomes, and may be useful for developing more culturally sensitive approaches to stress-related therapies.

## 1 Introduction

### 1.1 Stress as a cultural and psychological phenomenon

Stress is a complicated psychophysiological reaction and is the essential process of human adaptation and survival. Acute stress reactions allow individuals to rapidly activate cognitive, emotional and physiological resources to deal with environmental problems, but persistent or excessive stress can lead to major disruptions in physical and mental health (Lupien et al., 2009; McEwen and Stellar, 1993). Stress has been shown to be associated with dysfunction in many biological systems including the endocrine, immune, cardiovascular and nervous systems and has been found to be highly correlated with psychiatric disorders such as anxiety, depression and post-traumatic stress disorder (PTSD) (Dickerson and Kemeny, 2004; O’Connor et al., 2021).

Stress is not merely a biological response to objective stimuli in the environment per se. An increasing body of evidence points to the fact that the perception of stress, appraisal, emotional reactions, coping strategies, and recovery processes are strongly conditioned by sociocultural contexts (Chung et al., 2016; Taylor, 2006). People from different cultural backgrounds may interpret stressful situations, regulate emotions, seek support, and maintain interpersonal relationships under stress in very different ways. Thus, understanding stress from a cultural perspective is an important topic in cultural psychology and mental health research.

The influence of culture is notably relevant to PTSD. Individuals differ substantially in terms of vulnerability, symptom presentation and course of recovery after traumatic experiences such as war, violence, disasters, and major accidents (Yehuda, 2002). Previous studies have shown the relevance of cultural variables in trauma evaluation, autobiographical memory, coping mechanisms, use of social support, and meaning-making processes, all of which are directly associated to PTSD symptoms and adaptability (Jobson, 2009; Jobson et al., 2022). These processes are closely linked to how individuals understand themselves in relation to others and to their social world. From this perspective, self-construal may influence how traumatic events are interpreted, how trauma-related emotions are regulated, how social support is sought or avoided, and how traumatic experiences are integrated into one’s sense of self. Empirical studies have also begun to examine the association between self-construal and PTSD in trauma-exposed populations, including internally displaced persons and refugees with PTSD (Liddell et al., 2024; Mordeno et al., 2016). However, existing findings remain scattered across studies of acute stress, chronic stress, PTSD and cultural neuroscience. Therefore, an integrative review that spans multiple domains may help clarify how culturally formed self-processes, especially self-construal, are related to stress responses, adaptation, and PTSD-related processes.

### 1.2 Self-construal as a culture-shaped self-system

One of the most influential constructs connecting culture and self-related processes is self-construal. Self-construal refers to how individuals define, understand, and interpret themselves in relation to others within a sociocultural context (Cross et al., 2011; Markus and Kitayama, 1991). Two major types of self-construal have been proposed, namely independent self-construal (IndeSC) and interdependent self-construal (InterSC). Individuals with high IndeSC tend to define the self in terms of personal attributes, internal thoughts, and individual goals, whereas individuals with high InterSC tend to define the self in terms of social relationships, group memberships, and contextual roles (Markus and Kitayama, 1991). Importantly, these two forms of self-construal should not be understood as fixed cultural traits or as a simple East–West dichotomy, because both dimensions may coexist within individuals and may vary across social contexts.

Although both dimensions may be present within individuals, different cultural contexts tend to highlight one over the other (Oyserman et al., 2002). Collectivistic cultural contexts tend to emphasize social harmony, relational obligations, and interdependence, whereas individualistic cultural contexts tend to emphasize autonomy, self-expression, and personal achievement. Accordingly, self-construal has been widely regarded as an important psychological mechanism through which cultural contexts shape cognition, emotion, motivation, and behavior.

### 1.3 Why self-construal may shape stress responses

Previous research has shown that self-construal is associated with important cognitive, emotional, and motivational processes that are highly relevant to stress responses. At a cognitive level, high InterSC individuals are more sensitive to social context and interpersonal evaluation, whereas high IndeSC individuals may be more concerned with personal autonomy and self-consistency (Cross et al., 2011). As social-evaluative threat is considered as a key component of psychosocial stress (Dickerson and Kemeny, 2004), self-construal differences may influence the perception and appraisal of stressful situations. Emotionally, InterSC has been associated with a heightened sensitivity to socially engaging emotions such as shame and sympathy, whereas IndeSC is more associated with self-focused emotions such as pride and anger (Markus and Kitayama, 1991). Such emotional tendencies also might influence the strategies used for emotional regulation under stress. Motivationally, individuals with different self-construal orientations might select different coping strategies and regulatory goals when facing stress. For example, individuals with high InterSC are more likely to pursue relational harmony and self-adjustment, whereas those with high IndeSC might choose direct self-expression and primary control strategies (Oyserman et al., 2002).

Recently, researchers have started to explore neural correlates of self-construal and how they may relate to stress-related processes. Neuroimaging studies have revealed associations between self-construal and brain areas involved in self-r eferential processing, social cognition, emotional regulation and value-based decision-making, such as the medial prefrontal cortex (mPFC), temporoparietal junction (TPJ) and orbitofrontal cortex (OFC) (Han and Northoff, 2009; Kitayama et al., 2017). These brain areas have also been implicated in stress appraisal and emotional regulation. Although the current evidence is still preliminary and largely correlational, these findings raise the interesting possibility that culturally shaped self-processes may be related to stress responses not only at the psychological level but also at the neurobiological level.

Taken together, these results suggest that self-construal may influence the perception of stress, regulation of emotions, coping strategies, recovery from stress, and susceptibility to stress-related disorders such as PTSD.

### 1.4 Gaps in the literature

Although there has been growing interest in self-construal and stress, several important gaps in the literature remain. First, existing findings on the relation between self-construal and stress responses are highly fragmented across different domains, including acute stress, chronic stress, and PTSD. Second, prior studies have tended to focus on discrete psychological or physiological outcomes, without integrating cognitive, emotional, motivational, and neurobiological perspectives.

Third, although cultural and self-related factors have been discussed in PTSD research, the role of self-construal in stress-related disorders has not been systematically synthesized. In addition, some existing interpretations remain conceptually inconsistent regarding whether InterSC functions primarily as a protective factor or a vulnerability factor under stress-related conditions. Furthermore, many discussions regarding cultural differences in stress responses remain highly generalized and insufficiently integrated with contemporary cultural psychology frameworks.

Therefore, there is a need for a more comprehensive and culturally contextualized review that integrates evidence from cultural psychology, stress research, neuroscience, and PTSD-related studies.

### 1.5 Goal of this review

The goal of the present narrative review is to integrate the existing evidence on the association between self-construal and stress-related processes (i.e., acute stress, chronic stress, and PTSD) from cultural, psychological, and neurobiological perspectives. Specifically, this review will focus on how different types of self-construals may influence stress perception, emotional regulation, coping, stress recovery, and susceptibility to stress-related disorders.

This review brings together findings from cultural psychology, neuroscience and mental health research to provide a more holistic understanding of stress in a culturally contextualized framework and to discuss the potential implications for culturally sensitive psychological interventions.

### 1.6 Literature search strategy

The present article was conducted as a structured narrative review rather than a systematic review or meta-analysis. The main purpose was to provide a conceptual synthesis of representative theoretical and empirical studies linking self-construal to acute stress, chronic stress, and PTSD-related processes.

The review was guided by the following research question: How are InterSC and IndeSC associated with acute stress, chronic stress, and PTSD-related outcomes, and what cognitive, emotional, motivational, social, and neurobiological mechanisms may underlie these associations?

The literature included in the present review was identified through searches in several major academic databases, including PubMed, Web of Science, PsycINFO, and Google Scholar. The search was last updated on July 5, 2026. The searches mainly focused on studies related to self-construal, stress, culture, and PTSD. Search keywords included combinations of “self-construal,” “independent self-construal,” “interdependent self-construal,” “stress,” “acute stress,” “chronic stress,” “PTSD,” “cortisol,” “culture,” “social support,” “emotion regulation” and “neurobiology.”

The literature search identified approximately 200 records across databases. After removing duplicates and clearly irrelevant articles, approximately 70 studies were retained for full-text review. Around 50 empirical and theoretical studies directly relevant to self-construal and stress-related processes were ultimately included in the final synthesis. Additional foundational papers were identified through backward citation searching and theoretical relevance.

Studies were included if they met one or more of the following criteria: (1) they examined independent or interdependent self-construal directly; (2) they investigated cultural or self-related processes relevant to acute stress, chronic stress, or PTSD; (3) they contributed to understanding cognitive, emotional, motivational, social, or neurobiological mechanisms linking self-construal to stress-related outcomes; or (4) they provided foundational theoretical background for self-construal, stress, or PTSD. Studies were excluded if they focused on unrelated cultural variables without reference to self-processes, if they did not address stress- or trauma-related outcomes, or if they were conference abstracts, or unpublished manuscripts.

The search strategy was intended to identify representative and influential studies relevant to the review question rather than to achieve exhaustive coverage of all available publications. Because this is a narrative review, no formal PRISMA workflow, risk-of-bias assessment, or quantitative synthesis was conducted. Instead, the included literature was organized conceptually around three main domains: acute stress, chronic stress, and PTSD. Within each domain, we focused on how self-construal may influence stress-related processes through cognitive, emotional, motivational, social, and neurobiological pathways.

### 1.7 Scope of the review

The literature on self-construal is extensive. However, the present review does not aim to provide an exhaustive review of all studies on InterSC and IndeSC. Instead, it focuses on studies that are directly relevant to stress-related processes and PTSD. Foundational studies on self-construal are discussed to define the construct and to explain its cognitive, emotional, and motivational implications, whereas the main synthesis focuses on evidence linking self-construal to acute stress, chronic stress, trauma-related processes, and PTSD.

The number of studies directly examining self-construal in relation to stress and PTSD remains limited. For this reason, the review integrates direct empirical evidence with related findings from cultural psychology, emotion regulation, social support, autobiographical memory, and neurobiological studies. This approach allows the article to develop an integrative framework while also acknowledging that some proposed pathways remain preliminary and require further empirical testing.

## 2 The background and characteristics of self-construal

### 2.1 Conceptualization of self-construal

Self-construal describes how individuals make sense of the self in relation to others and to the surrounding social context. Markus and Kitayama (1991) proposed two broad orientations of self-construal: the IndeSC and the InterSC. IndeSC defines the self independently of social contexts; they emphasize antonomy, uniqueness, self-expression and personal goals. InterSC see themselves as connected to others; they emphasize connectedness, social roles, relational harmony, and adjustment to others. These orientations are not mutually exclusive. Rather, they represent psychological tendencies that may be shaped by cultural contexts and activated differently across interpersonal situations. Thus, self-construal provides a useful framework for understanding how culture becomes linked to individual cognition, emotion, motivation, and behavior.

### 2.2 Cognitive characteristics of self-construal

Cognitive processes play an important role in shaping and influencing the self, vice versa, different characteristics of the self influence cognitive processes. Individuals with high IndeSC perceive the self with more emphasis on independence and differentiation, are less dependent on the surrounding social environment to define the self, and accordingly show less environmental sensitivity. In contrast, individuals with InterSC have more relational, collective, and social descriptions of self. This is because in a social environment dominated by a collectivist culture, maintaining relationships and good social interactions requires a full understanding of how others think and feel. InterSC formed in such environments naturally recognize and understand the self and others in the context of society, and is more environmentally sensitive (Cross et al., 2011) as well as more likely to connect with others and be assimilated into the larger environment. Individuals with InterSC often have more detailed cognitive representations of others than of themselves. In contrast, individuals with IndeSC typically have more unique and detailed perceptions of the self. These cognitive differences might be relevant for the processing of stress. Increased sensitivity to social evaluation and contextual information may lead to a higher vulnerability in socially threatening situations, whereas increased self-focused processing may enable more independent coping strategies.

### 2.3 Emotional characteristics of self-construal

Self-construal also affects emotional experience and emotional expression. Previous cultural psychological theories have suggested that high IndeSC and InterSC differ not only in the emotions that they experience more frequently but also in the interpretation and expression of emotions (Markus and Kitayama, 1991).

People with high IndeSC tend to experience and display more ego-focused and socially disengaging emotions, such as pride, frustration, and anger, which are often related to personal achievement, self-esteem, and individual goal pursuit. In terms of subjective well-being, high IndeSC is thus more strongly associated with self-consistency, autonomy, and personal emotional experience.

In contrast, people high in InterSC are more likely to experience socially engaging and other-focused emotions such as sympathy, shame, interpersonal closeness, and relational concern. Interpersonal harmony and social connectedness are highly valued, and emotional experiences are often tightly linked to social relationships and perceived evaluations from others. People high in InterSC are also generally more sensitive to interpersonal feedback, and may place greater emphasis on regulating emotional expression to maintain relational harmony.

Several studies have indicated that InterSC is associated with increased levels of social anxiety, maladaptive perfectionism and depressive symptoms while IndeSC is often associated with increased subjective happiness and decreased levels of some of the negative emotions (Cross et al., 2011). These emotional characteristics are highly relevant to stress processes, especially because acute and chronic stress often involve emotional appraisal, interpersonal evaluation, and emotion regulation.

### 2.4 Motivational characteristics of self-construal

Self-construal also affects motivational orientation and coping behavior. Individuals with high IndeSC tend to be more driven by personal success, self-development and self-expression. Their aims generally are tied to achieving personal strengths, expressing individual choices, and sustaining autonomy (Oyserman et al., 2002). In contrast, individuals with high InterSC are more relational goal-oriented and socially obligated. They tend to value harmonious relationships, social roles, and the expectations of others over individual distinctiveness. Importantly, this does not mean that people high in InterSC have a higher overall need to belong, but rather that they are more motivated to maintain important interpersonal relationships and social connectedness.

Differences in self-construal are also associated with different coping strategies. High IndeSC people are more likely to take main control strategies to change the external environment to meet their needs. High InterSC people, in contrast to low InterSC people, usually use secondary control strategies more often in an effort to control themselves and adapt themselves to environmental demands by changing their internal feelings, attitudes and perceptions.

These motivational differences may have different effects on stress responses. Greater sensitivity to interpersonal expectations may increase the perceived burden of social evaluation and role responsibilities, whereas greater self-focused motivation may facilitate autonomous coping and emotional expression. However, the secondary control strategies more frequently employed by the InterSC individuals may also be adaptive in collectivist settings, and persistent self-regulation and emotional repression may lead to the build-up of chronic stress.

### 2.5 Neural basis of self-construal

Previous studies have demonstrated that differences in self-construal are linked to both functional and structural differences in the brain, particularly in brain regions involved in self-referential processing, social cognition, emotion regulation, and value-based decision-making.

The medial prefrontal cortex (mPFC), an important area involved in self-referential processing, has been repeatedly associated with independent self-related cognition (Han and Northoff, 2009). Using functional magnetic resonance imaging (fMRI), Ma et al. (2014) found that people with a higher level of IndeSC showed a greater mPFC activation during self-judgment activities. In contrast, greater InterSC was associated with increased temporoparietal junction (TPJ) activation, but decreased mPFC activation during self-reflection on social attributes (Han and Humphreys, 2016; Ma et al., 2014).

Structural neuroimaging study has further associated self-construal with changes in the volume of the orbitofrontal cortex (OFC). In fact, Kitayama et al. (2017) found that greater InterSC was related to smaller OFC volumes. The OFC has been implicated in value-based decision making, emotional regulation, and stress-related appraisal processes (Ansell et al., 2012; Seo et al., 2011). Recent stress research has similarly shown associations between smaller OFC volume and greater subjective experience of stress (Ansell et al., 2012) and greater OFC activation during acute stress and greater stress responses (Seo et al., 2011).

Overall, these findings suggest a potential neurobiological basis for the link between self-construal and stress-related processes. In a fMRI study (Luo et al., 2023), a positive association was found between the level of InterSC and OFC activation in response to acute psychosocial stress, which raises the possibility that self-construal may be related to neural systems involved in social appraisal and emotional processing under stress. However, current findings are mostly correlational and do not demonstrate that self-construal directly causes specific structural or functional brain changes. Therefore, the neural findings reviewed here should be viewed as preliminary evidence and as hypotheses for future research rather than as established causal mechanisms.

## 3 Acute/chronic stress and PTSD

Acute stress, chronic stress, and post-traumatic stress disorder (PTSD) are different temporal and severity expressions of stress responses, but have a commonality in that all involve coordinated cognitive, emotional, physiological, and behavioral adaptations to environmental demands. Instead they can be viewed as a continuum of stress-related processes from short-term adaptive reactions to long-term detrimental results rather as independent categories.

Acute stress is a short-term response to acute environmental demands such as evaluative tasks, physical risks or social stresses. This reaction is mediated primarily by fast activation of the sympathetic-adrenal-medullary (SAM) system followed by engagement of the hypothalamic–pituitary–adrenal (HPA) axis (Dickerson and Kemeny, 2004; McEwen, 2007). These neuroendocrine systems make possible short-term adaptation, mobilizing energy resources and increasing attention. Importantly, acute stress is not necessarily bad. In fact, the functional importance of stress is in its dynamic management, including response and recovery.

Chronic stress, on the other hand, is a state of repeated or prolonged exposure to stressors over an extended period of time without sufficient recovery, resulting in the accumulation of allostatic load and increased physiological wear and tear (McEwen, 1998; McEwen and Gianaros, 2011). Chronic stress is also less characterized by discrete events and more so by continuous cognitive-emotional load, commonly rooted in interpersonal, occupational, or identity-based contexts (Cohen et al., 2012). Chronic activation of stress-related systems has been associated with dysregulation of cortisol cycles, and increased hair cortisol concentration (Lupien et al., 2009).

Stress can occur from exposure to trauma including violence, tragedy, or serious threat and may result in the development of PTSD, which is a stress-related disorder involving the persistence of re-experiencing the traumatic event, avoidance, hyperarousal, and unfavorable modifications in cognition and heightened arousal that persists for more than one month (Pitman et al., 2012). Addtionally, PTSD represents a breakdown of normal recovery and integration processes following exposure to intense stress, and suggests abnormalities in both memory processing and self-related cognitive frameworks (Jobson, 2009; Yehuda, 2002).

These three types of stress differ not just in their timescale but also in the degree to which cognitive assessment, emotional regulation, and self-referential processing are involved conceptually. Acute stress involves fast evaluation and immediate physiological mobilization. Chronic stress is a state of extended cognitive-emotional involvement with persistent demands. PTSD, in turn, is characterized by maladaptive rearrangement of autobiographical memory and self-identity following trauma exposure (Brewin, 2011).

This distinction is of relevance for the present review. Self-construal is unlikely to affect only one sort of stress reaction. Rather, it could function on different levels of the stress system, affecting the appraisal of stressors, the generation and regulation of emotional responses, and the recovery or integration of stressful experiences over time. Therefore, rather than conceptualizing acute stress, chronic stress and PTSD as independent topics, this review approaches them as interrelated consequences of stress processing that might be variably modulated by culturally influenced self-construal.

## 4 Effects of self-construal on acute/chronic stress and PTSD

Self-construal may affect stress-related processes at multiple stages, from the initial appraisal of stressors and acute physiological activation to the prolonged accumulation of effects and adaptation to trauma. Individuals with high InterSC may be more sensitive to social-evaluative cues, show greater acute stress reactivity, and be more vigilant in interpersonal situations. Over time, persistent effect of these processes may contribute to chronic stress burden and potentially influence vulnerability to PTSD-related symptoms. The following sections review evidence across these interconnected levels of stress processing.

### 4.1 The association between self-construal and stress

Stress is a comprehensive response of an individual that integrates cognitive, emotional, and motivational mental processes. Firstly, cognition determines how individuals perceive and evaluates the stressor before and during the onset of stress (Lazarus, 1984); meanwhile individuals’ emotion regulation strategies such as inhibiting expression can alleviate the effects of stress. Motivation to achieve a certain goal may alleviate or exacerbate stress, e.g., overly high personal goals and expectations can increase stress, especially if they are difficult to achieve (Hobfoll, 1989; Ryan and Deci, 2000). Secondly, stress exposure is often accompanied by elevated anxiety, frustration, and negative affect. The effects of stress on motivation seem more complex - whereas acute stress may increase motivation to engage in a challenge (McEwen, 2008), long-term stress can drain psychological and physiological resources, resulting in burnout, fatigue and decreased motivation (Maslach and Leiter, 2016).

In addition to playing an important role in the stress response, cognition, emotion, and motivation are often involved in the process of stress coping. Previous research has shown that changing cognitive appraisals improves the effect of coping with stress and reduces stress reactions, improves psychological functioning (Gross, 2002; John and Gross, 2004). Emotionally focused coping has been shown to be effective in managing emotional distress and maintaining life well-being in the face of stressors that are completely out of the individual’s control (Folkman and Moskowitz, 2004). Being motivated usually means having a need, and studies have shown that satisfying individuals’ basic psychological needs (autonomy, competence, relationships) can effectively cope with stress and enhance well-being (Ryan and Deci, 2000).

Overall, the process of stress and coping with stress involves a range of psychological and physiological responses in which an individual’s cognition, emotion, and motivation all play important roles. Combined with the previous discussion of self-construal, both IndeSC and InterSC have their own cognitive, emotional, and motivational characteristics. We speculate that self-construal, a culturally based conceptualization of the self, is likely to influence stress reactions as well as coping with stress. Stress is closely related to physical and mental health. The study combining self-construal with stress and stress coping can theoretically expand the understanding of stress from the perspective of cultural context and self, and practically apply cultural context and self to the field of health, which provide people a new perspective to cope with stress, and promote physical and mental health. Stress-related processes are divided into acute and chronic stress based on criteria including duration and severity (Chida and Hamer, 2008), and these are discussed in the following sections.

### 4.2 The effect of self-construal on the acute stress response

Acute stress induces a series of physiological and psychological responses, including increases in cortisol secretion, heart rate, blood pressure, and subjective feelings of stress. Salivary cortisol is one of the most extensively utilized objective markers of hypothalamic–pituitary–adrenal (HPA) axis activation in the context of psychosocial stress (Dickerson and Kemeny, 2004). Previous research on self-construal and acute stress has largely employed paradigms of social-evaluative threat, including the Trier Social Stress Test (TSST), Montreal Imaging Stress Task (MIST), and Socially Evaluated Cold Pressor Test (SECPT). Table 1 summarizes previous studies investigating the association between self-construal and acute stress response. Although the current empirical literature remains limited and several studies originate from the same research line, the available findings show relatively consistent associations between InterSC and heightened psychosocial stress responses.

**Table 1:** Summary of studies on relationship between InterSC and acute stress response.

Literature	Sample size	Self-construal measurement	Acute stress Paradigm	Index of acute stress response	Main results
Hu et al. (2018)	*n* = 46	SCS	TSST	Subjective stress Heart rate Salivary cortisol	The salivary cortisol level, subjective stress and heart rate of the high InterSC group were significantly higher than those of the low InterSC group.
Hu et al. (2019)	*n* = 55	SCS	SECPT	Social evaluative threat Subjective stress (AUCi) Salivary cortisol (AUCi)	InterSC was positively correlated with social evaluative threat and salivary cortisol ACUi; InterSC was not significantly correlated with subjective stress.
Ren et al. (2019)	*n* = 55	SCS	TSST	Subjective Stress (AUCi) Heart rate (AUCi) Salivary cortisol (AUCi)	Levels of InterSC positively predicted cortisol increase in stressful situations. Individuals with high InterSC had lower salivary cortisol increase after experiencing social support compared to self support.
He et al. (2021)	*n* = 84	SCS	TSST	Subjective stress (change rate) Salivary cortisol (change rate)	High InterSC individuals showed a pattern of fast response and fast recovery in both cortisol response and subjective stress during acute stress.
Luo et al. (2023)	*n* = 44	SCS	MIST	Subjective stress (AUCg, AUCi) Salivary cortisol (AUCg, AUCi)	InterSC was positively correlated with subjective stress AUCg, but was not significantly correalted with salivary cortisol AUCg and AUCi.
Luo et al. (2024)	*n* = 73	SCS	ScanSTRESS	Social Anxiety Stress reactivity slope Stress recovery slope Salivary cortisol (AUCg, AUCi)	InterSC was positively correlated with social anxiety, but was neither significantly associated with cortisol response AUCg nor AUCi. High InterSC showed a rapid reactivity and recovery of salivary cortisol during acute stress.

SCS, self-construal scale; TSST, trier social stress test; SECPT, social evaluative cold pressor test; MIST, montreal imaging stress task; AUCg, the area under the curve with respect to ground; AUCi, the ares under the curve with respect to increase; EE, emotional empathy.

InterSC has been found to be related to greater psychosocial stress responses across research, including greater subjective stress, cortisol reactivity, and heart rate (Hu et al., 2019, 2018; Luo et al., 2023; Ren et al., 2019, Talavera et al., 2019). One possibility is that those high in InterSC see the self in relation to others and are hence more sensitive to interpersonal judgment, social criticism, and relational discord. Social-evaluative threat is considered one of the strongest elicitors of acute stress responses (Dickerson and Kemeny, 2004) and high InterSC may be related to more stress activation in socially evaluative contexts.

It is important to note that the link between InterSC and acute stress should not be interpreted as simply maladaptive. Some studies have indicated that highly InterSC individuals might exhibit a stronger stress reactivity but a relatively efficient recovery following stress exposure (He et al., 2021; Luo et al., 2024). In this way, InterSC might result in an increased sensitivity to social-environmental demands and at the same time, a quick adaptation when the stressor is eliminated. Such a tendency may reflect adaptation to collectivistic cultural situations in which social attunement and interpersonal harmony are highly valued.

However, the adaptive significance of this increased sensitivity may rely on contextual and regulatory factors. Moderate increases in stress reactivity may allow people to mobilize cognitive and social resources in difficult situations, but excessive or prolonged activation may raise vulnerability to anxiety, chronic stress accumulation, or stress-related disorders. Hence, InterSC can be a protective and risk factor depending on the severity, duration and social relevance of stress exposure.

### 4.3 Acute stress recovery and social adaptation

The stress response is a dynamic process, consisting of two main phases: reaction and recovery (Linden et al., 1997; Roy, 2004). Stress can affect your health in two ways. Moderate stress has a protective effect, individuals are in a situation to make a rapid “fight or flight” decision, which is conducive to coping with crises and boosts adaptive capacity. However, when the stress reaction exceeds the capacity of regulation, it may lead to persistent and major physiological activity abnormalities and eventually cause medical and mental illnesses (McEwen and Stellar, 1993; O’Connor et al., 2021). Recovery after stress is essential, as prolonged physiological activation may contribute to allostatic load and increase the risk of physical and mental health problems (McEwen, 1998).

Interestingly, previous studies in collectivistic cultural contexts have shown that individuals high in InterSC exhibited larger initial stress reactions, as well as a faster rate of recovery in subjective stress and cortisol responses (He et al., 2021; Luo et al., 2024). One reason might be that those high in InterSC are more accustomed to monitoring social cues and adjusting to interpersonal demands. This enhanced sensitivity may be advantageous for the initial amplification of stress reactions in evaluative circumstances, but may also permit rapid recalibration once social risk is reduced. Viewed in this way, high sensitivity coupled with rapid recovery may be an adaptive regulating style, not vulnerability alone. But this adaptive interpretation should be accompanied by caution. Thus far, evidence is limited and most findings are based on East Asian collectivistic conditions. More cross-cultural study is needed to determine when InterSC increases resistance or vulnerability to stress.

Acute stress recovery may also influence subsequent socioemotional performance. Empathy after acute stress has been studied with inconsistent findings, showing that stress can improve, hinder or not influence empathic reactions depending on contextual and individual variables (Buruck et al., 2014; Wingenfeld et al., 2018; Wolf et al., 2015). It is speculated that self-construal may play a role in this, but further confirmation is still needed.

### 4.4 Social support intervention

Excessive stress can be detrimental to an individual’s physical and mental health, one important strategy to regulate and alleviate stress is social support. Social support is the important information that convinces individuals that they are loved, cared for, respected, and accepted by the group (Cobb, 1976). Social support, as a manifestation of important individual-individual and individual-group dependencies, influences an individual’s ability to face challenging and stressful events (Caplan, 1981), and those individuals who have good social support exhibit lower levels of cortisol stress (Heinrichs et al., 2003; Kirschbaum et al., 1995; Ren et al., 2019). Hence people endeavor to make social connections with others, thereby reducing the potential risks that may be posed by stressors (Taylor, 2006). And the definition of InterSC itself implies connecting with others, which is manifested in the way individuals regulate themselves to adapt to and meet the needs and expectations of others, which can be exchanged for others’ recognition and support. Therefore, based on this characteristic of the InterSC, we speculated that although previous findings suggested that individuals with high InterSC have stronger stress reactions, social support is an effective strategy to alleviate stress in them. The results suggest that compared to self support, individuals with high InterSC who experience social support and then face an acute stress do exhibit lower amounts of elevated cortisol (Ren et al., 2019). One possible explanation is that interpersonal support fits well with the relational orientation associated with InterSC. In many collectivistic cultural contexts, feelings of safety and stress regulation are often closely tied to close relationships and a sense of belonging to social groups (Boroş et al., 2019; Čavojová et al., 2022).

However, individuals with high InterSC may not always actively look for support; they may be less inclined to directly seek help due to concerns about burdening others or disturbing interpersonal harmony (Lee et al., 2015; Taylor et al., 2020). In this sense, InterSC may increase the importance of social support while at the same time discouraging direct support-seeking behaviors. This pattern has been reported repeatedly in East Asian cultural contexts.

It is important to note that the role of social support may vary across sociocultural contexts. Even in societies that place greater emphasis on independence, InterSC may still facilitate stress buffering through close interpersonal connections (Oyserman et al., 2002). Thus, the stress-buffering effects of social support may depend not only on cultural orientations but also on individual differences in relational orientation.

### 4.5 The effect of self-construal on chronic stress level

Chronic stress is a reaction that occurs when an individual is exposed to low to moderate stressors over an extended period of time. Unpleasant interpersonal interactions or interpersonal strife, disturbing work, financial stress, criticism or blame from others, and challenges to one’s identity are often important causes of chronic stress (Cohen et al., 2012; Marin et al., 2011). Studies examining the relationship between self-construal and chronic stress are summarized in Table 2. The results showed that InterSC has been associated with higher perceived daily stress and physiological markers of long-term stress regulation such as cortisol awakening response (CAR) and hair cortisol concentration (HCC) (Burt et al., 2018; Cross, 1995; Hu et al., 2018).

**Table 2:** Summary of studies on relationship between InterSC and chronic stress level.

Literature	Sample size	Self-construal measurement	Index of chronic stress response	Main results
Cross (1995)	East Asian students (*n* = 71) American students (*n* = 79)	Ego-tasks	PSS	Among East Asian students, individuals with high InterSC perceived more stress.
Burt et al. (2018)	*n* = 316	SCS	CAR	InterSC was positively correlated with CAR in first generation Asian American students but not the second generation.
Hu et al. (2018)	Study 1 (*n* = 295) Study 2 (*n* = 46)	SCS	Study 1: DSI Study 2: CAR	Study 1: InterSC is positively correlated with DSI scores. Study 2: The CAR AUCi in high InterSC group is significantly higher than that in low InterSC group.
Talavera et al. (2019)	*n* = 176	SCS	PSS	Among women from ethnic minority groups, lower IndeSC was associated with greater perceived stress.
Luo et al. (2024)	*n* = 73	SCS	HCC	InterSC positively correlates with HCC, with social anxiety mediating this association.

SCS, self-construal scale; CAR, cortisol awakening response; PSS, perceived stress scale; DSI, daily stress inventory; HCC, hair cortisol concentration.

In line with this, subjective measures of chronic stress such as the Daily Stress Inventory (DSI) and Perceived Stress Scale (PSS) consistently indicate that those high in InterSC tend to report higher levels of daily stress (Cross, 1995; Hu et al., 2018). One possible explanation is that individuals who have a high InterSC might be more concerned about interpersonal harmony, social evaluation, and obligations in relationships. This could result in being more sensitive to social-environmental demands.

Physiological findings demonstrate a similar tendency. CAR, which reflects the rapid increase of the cortisol secretion after awakening (Pruessner et al., 1997), has been associated with the regulation of chronic stress and anticipatory adaptation to daily demands. Some studies have found a positive association between InterSC and CAR, particularly among first-generation Asian American students and individuals in collectivistic cultural contexts (Burt et al., 2018; Hu et al., 2018). Such findings may indicate that InterSC is linked to increased vigilance to predicted social and environmental threats. However, severe or chronic stress over a prolonged period of time can impair the systems that regulate cortisol (Fries et al., 2009), which suggests that the association between InterSC and chronic stress is unlikely to be uniformly adaptive.

More recently, HCC has been used more frequently as an objective biomarker of long-term stress exposure, as it is indicative of cumulative cortisol secretion over longer periods of time (Russell et al., 2012). Preliminary results also found that InterSC was possibly positively related to HCC, perhaps mediated by social anxiety. However, HCC is affected by many environmental and behavioral factors, and the current findings are still somewhat limited. Hence, more longitudinal and cross-cultural research is necessary to elaborate the association between self-construal and regulation of chronic stress.

### 4.6 The effect of self-construal on PTSD

PTSD is a trauma-related disorder that involves intrusive memories, avoidance, hyperarousal and negative alterations in cognition and mood following exposure to traumatic experiences (Brewin et al., 2010; Yehuda, 2002). There is growing evidence that PTSD is not only influenced by the traumatic event itself, but also by trauma appraisal, autobiographical memory, emotion regulation, interpersonal context, and self-related cognition (Jobson, 2009; Jobson et al., 2022). These processes are closely related to self-construal, since self-construal shapes how individuals understand themselves, others, and social relationships.

From the perspective of self-construal, trauma may threaten different aspects of the self. For individuals with higher IndeSC, trauma-related distress may be closely related to perceived loss of personal control, autonomy, competence, or self-consistency. For individuals with higher InterSC, traumatic distress may be more strongly connected to relational disruption, shame, social evaluation, family responsibility, or perceived failure to meet interpersonal expectations. These possibilities do not imply that one form of self-construal is inherently protective or harmful. Rather, they suggest that self-construal may shape the meaning of trauma and the pathways through which PTSD symptoms develop or are maintained.

Several mechanisms may link self-construal to PTSD-related outcomes. First, self-construal may influence trauma appraisal. Individuals with high IndeSC may be more likely to evaluate trauma in relation to personal agency and self-efficacy, whereas those with high InterSC may be more sensitive to relational consequences and social evaluation. Second, self-construal may influence autobiographical memory. Because PTSD involves difficulties in integrating traumatic memories into the broader self-system, cultural differences in self-related memory organization may affect how trauma is remembered and narrated. Third, self-construal may shape coping and emotion regulation. Individuals high in InterSC may be less willing to express distress openly because of concerns about burdening others or disturbing relational harmony. This may increase avoidance or emotional suppression in some contexts, but it may also facilitate recovery when relational support is available and culturally appropriate.

Recent empirical evidence supports these proposed mechanisms. Jobson et al. (2025) found that cultural background and self-construal jointly influenced the association between expressive suppression and PTSD symptoms. Specifically, the positive association between expressive suppression and PTSD symptoms was attenuated among trauma survivors who endorsed higher levels of interdependent self-construal, suggesting that the effects of emotion regulation strategies may vary across cultural and self-related contexts. Similarly, Li et al. (2025) reported that interdependent self-construal moderated the associations between multiple forms of rumination and PTSD symptoms. Individuals with higher levels of interdependence showed a stronger relationship between rumination and PTSD symptom severity, highlighting the potential role of culturally shaped self-processes in trauma-related cognitive vulnerability.

Additional evidence comes from trauma-exposed populations. Mordeno et al. (2016) examined PTSD symptom structure and its relationship with self-construal among internally displaced persons, suggesting that self-related cultural processes may be relevant for understanding PTSD vulnerability and adaptation following trauma. More recently, Liddell et al. (2024) demonstrated that PTSD and self-construal interacted to influence default mode network (DMN) connectivity, particularly in the ventromedial prefrontal cortex (vmPFC). Given the role of the DMN in self-referential processing, autobiographical memory, and identity-related processes, these findings provide preliminary neurobiological support for theoretical models proposing a role for self-construal in PTSD-related processes.

These findings point to the potential value of culturally sensitive approaches to PTSD intervention. Individuals with stronger IndeSC may benefit from interventions that emphasize agency, autonomy, personal control, and self-efficacy, whereas individuals with stronger InterSC may benefit from approaches that emphasize relational support, social reconnection, family meaning, and culturally appropriate ways of expressing distress. At the same time, empirical research directly examining self-construal and PTSD remains limited. More longitudinal, clinical, and cross-cultural studies are needed to clarify how self-construal influences trauma adaptation and treatment response.

## 5 Discussion

This review evaluated the association of self-construal with stress-related processes, such as acute stress, chronic stress, and PTSD, from a cultural viewpoint. In short, the results indicate that self-construal is directly linked to the way individuals perceive, react to, regulate and recover from stress. The available information suggests that InterSC is typically linked to elevated sensitivity toward social-evaluative stresses, subjective stress perception, and physiological stress responses. At the same time, InterSC may also assist faster stress recovery and increased reactivity to social support in particular cultural circumstances. These findings imply that self-construal may influence patterns of stress management through cognitive assessment, emotional processing, coping style, and interpersonal orientation.

The present review also emphasizes the relevance of understanding stress within a cultural context. Stress responses are a function not only of the objective strength of stressors but also of individuals’ interpretations of social interactions, evaluations of the self in relation to others, and regulation of emotions within culturally established value systems. Thus, self-construal offers a valuable paradigm to link culture, self-processes and stress-related mental health consequences.

In sum, the research discussed here provides an integrated framework indicating how culturally formed self-construal could affect stress-related processes (see Figure 1). Specifically, IndeSC and InterSC may lead to differential effects on cognitive evaluation, affective processing, and motivational orientation that may, in turn, influence acute stress response, control of chronic stress, and susceptibility to PTSD. And these linkages may also be further affected by social support and neurological mechanisms. Although the research is mostly correlational, the framework provides a culturally contextualized lens through which to examine how self-related processes may impact stress adaptation and mental health outcomes.

**Figure 1:**
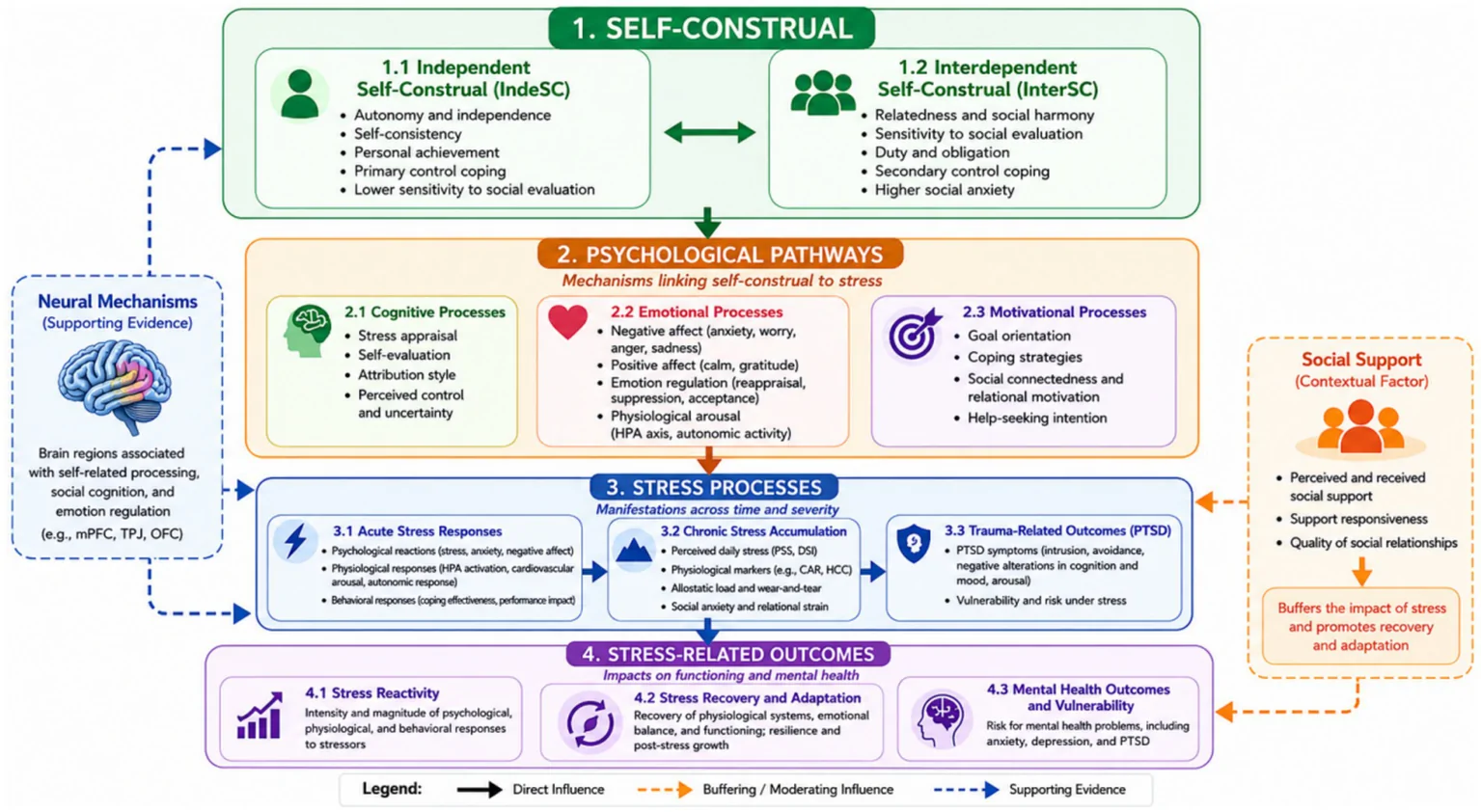
Integrative framework of the associations between self-construal and stress-related processes.

Notably, the current literature appears to show more consistent associations between stress-related processes and InterSC than IndeSC. One possible explanation is that many commonly used psychosocial stress paradigms, such as the TSST and other social-evaluative stress tasks, primarily involve interpersonal evaluation and social judgment, which may be particularly relevant to InterSC-related processes. However, the relatively limited findings regarding IndeSC should not be interpreted as evidence that independent self-related processes are unrelated to stress responses. Recent evidence further suggests that independent self-construal may also serve as a protective factor in certain stress-related contexts. For example, Wang et al. (2025) found that higher levels of IndeSC were associated with a weaker relationship between acculturative stress and maladaptive behavioral outcomes, suggesting that autonomy-related self-processes may buffer certain forms of chronic stress. Future research may benefit from examining stress contexts more closely related to threats to personal autonomy, self-consistency, and individual goal attainment.

### 5.1 InterSC and chronic stress

The studies addressed in this paper demonstrate that InterSC is often related with elevated levels of chronic stress, as reflected in subjective stress ratings, CAR, and HCC. One interpretation could be that people high in InterSC are more sensitive to social appraisal and interpersonal feedback. Stressors involving criticism, interpersonal conflict, role expectations, or relational instability may become more psychologically salient because the self is more strongly defined by relationships and social roles.

Cognitively, high InterSC individuals are more inclined to monitor social situations, and to analyze their impressions by others. Emotionally, they may experience more intense socially involved emotions such as shame, guilt, or a desire for interpersonal peace. InterSC is also more related to secondary control mechanisms in coping processes, such as self-adjustment and adaptability to environmental demands (Cross et al., 2011). However, these features may facilitate social coordination and relationship maintenance, but they may also lead to increased long-term psychological alertness and stress burden.

However, the association between InterSC and stress should not be viewed as purely maladaptive. Moderate levels of stress sensitivity might have adaptive value in collectivistic cultural contexts. Increased interpersonal sensitivity may help individuals to maintain social harmony, meet relational obligations, and respond more effectively to social demands. On this basis, InterSC increases sensitivity to socially relevant information, which may be adaptive under manageable social demands but maladaptive under prolonged or uncontrollable stress exposure. The effects of InterSC on chronic stress seem to depend on the balance between adaptive social control and extended psychological burden.

### 5.2 InterSC and acute stress regulation

Across the research reviewed here, high InterSC was consistently associated with heightened acute stress responses, particularly to psychosocial stress including social-evaluative threat. This was observed in subjective stress perception, cortisol response and heart rate variation in a range of experimental paradigms including the TSST, MIST and SECPT. Given the emphasis that InterSC places on connection to others and sensitivity to appraisal by others, socially evaluative situations may become especially salient stressors.

In addition, some studies also demonstrated that high InterSC individuals recovered quite quickly after acute stress exposure. They appeared to have a pattern of higher stress reactivity and faster post-stress recovery compared to low InterSC individuals. The current evidence implies that InterSC may not only augment stress responses, but rather may build a culturally linked stress regulation style marked by quick mobilization and efficient recovery in the context of socially relevant stress.

One possible reason is that individuals with high InterSC are more adapted to monitor interpersonal situations and to self-adjust according to social demands. Such long-term adaptation to socially embedded environments may increase initial stress sensitivity, but also facilitate recovery once the stressor has been eliminated. However, evidence to date is still sparse and mostly focused on samples from collectivist cultures. Additional studies are required to investigate whether similar effects exist in individualistic cultural situations.

The findings on social support further highlight the contextual importance of self-construal in regulating stress. Those with high InterSC may often be reluctant to actively seek help because they worry about burdening others, but social support appears to be very good at lowering their stress reactions once they receive support (Ren et al., 2019). This shows that the stress-buffering benefits of social support may depend not only on the availability of assistance but also on culturally molded interpersonal expectancies and self-related values.

### 5.3 Adaptive balance between IndeSC and InterSC

The relationship between IndeSC and InterSC should probably not be understood as a simple opposition. Although collectivistic cultural contexts tend to emphasize InterSC whereas individualistic cultural contexts tend to emphasize IndeSC, both dimensions may coexist within the same individual to varying degrees (Oyserman et al., 2002).

From an adaptive perspective, different combinations of self-construal may be associated with different patterns of stress regulation and social functioning. Individuals high in InterSC but low in IndeSC may be more socially sensitive and relationship-oriented, whereas those high in IndeSC but low in InterSC may place greater emphasis on autonomy and self-consistency. However, some people could have a reasonably high level of both the IndeSC and the InterSC simultaneously. Such individuals may value personal autonomy and interpersonal connectedness and so may be more flexible in balancing personal and social responsibilities.

Previous research has demonstrated that flexible or integrated self-construal may facilitate perspective-taking, social adjustment, and adaptive coping across circumstances (Cross et al., 2011). But in some settings, the tension between strong individual and relational needs could potentially exacerbate internal conflict and persistent psychological load. Thus, the adaptability of self-construal may be less dependent on whether people are essentially autonomous or interdependent, and more on their ability to flexibly regulate self-related orientations to satisfy the needs of the setting.

On the other side, individuals low on both IndeSC and InterSC could experience a less developed sense of personal identity and weaker social bonds. They may have less pressure from autonomy or relationship commitments but may encounter difficulties with belonging, motivation or social integration. There is still little empirical evidence on these trends and additional research is necessary.

### 5.4 Clinical implications and future directions

The present review has various implications for research and intervention in stress-related mental health. First, it underscores the need for adding culturally molded self-processes in our understanding of stress and PTSD. Self-construal affects assessment, coping, emotional regulation, and use of support. Culture-sensitive intervention strategies can make therapy more effective.

Individuals high in IndeSC may be more responsive to interventions that emphasize personal control, autonomy and self-efficacy, whereas high InterSC people may be more responsive to approaches that emphasize interpersonal connectedness, relational support and social integration (Jobson, 2009). Likewise, knowledge of cultural differences in help-seeking behavior may also improve social support programs for stress-related disorders.

There are also other limitations in the current literature that deserve mentioning. Current research studies are still restricted in sample variety and most conclusions are mostly centered on East Asian collectivist situations. More longitudinal and cross cultural studies are needed to clarify causal links between self-construal and stress control. Future studies should also further combine subjective, physiological and neurobiological indices to better understand the mechanisms relating culture, self-processes and stress-related effects.

Overall, the current research and the integrative framework proposed in Figure 1 suggest that self-construal may be a useful perspective for understanding how culturally molded self-systems influence stress responses, adaption and mental health consequences. Cultural psychology has a part to play in the study of stress, and can contribute to the development of more culturally sensitive models of emotional regulation and psychological adaptation.
